# The application of an age adjusted D-dimer threshold to rule out suspected venous thromboembolism (VTE) in an emergency department setting: a retrospective diagnostic cohort study

**DOI:** 10.1186/s12873-022-00736-z

**Published:** 2022-11-23

**Authors:** Liam Barrett, Tom Jones, Daniel Horner

**Affiliations:** 1 Emergency Department, Cambridge University Hospitals NHS Foundation Trust, Cambridge, CB2 0QQ UK; 2 University Division of Anaesthesia, Cambridge University, Cambridge, UK; 3 Wythenshawe Hospital, University of Hospital of South Manchester, Southmoor Road, Wythenshawe, M23 9LT UK; 4 Emergency Department, Salford Royal NHS Foundation Trust, Stott Lane, Salford, UK; 5 Division of Infection, Immunity and Respiratory Medicine, University of Manchester, Manchester, UK

**Keywords:** Venous thromboembolism (VTE), D-dimer, DVT, PE, Emergency medicine, Compression Ultrasonography

## Abstract

**Background:**

Venous Thromboembolic disease (VTE) poses a diagnostic challenge for clinicians in acute care. Over reliance on reference standard investigations can lead to over treatment and potential harm.

We sought to evaluate the pragmatic performance and implications of using an age adjusted D-dimer (AADD) strategy to rule out VTE in patients with suspected disease attending an emergency department (ED) setting. We aimed to determine diagnostic test characteristics and assess whether this strategy would result in proportional imaging reduction and potential cost savings.

**Methods:**

Design: Single centre retrospective diagnostic cohort study.

All patients > 50 years old evaluated for possible VTE who presented to the emergency department over a consecutive 12-month period between January and December 2016 with a positive D-dimer result. Clinical assessment records and reference standard imaging results were followed up by multiple independent adjudicators and coded as VTE positive or negative.

**Results:**

During the study period, there were 2132 positive D-dimer results. One thousand two hundred thirty-six patients received reference standard investigations. A total increase of 314/1236 (25.1%) results would have been coded as true negatives as opposed to false positive if the AADD cut off point had been applied, with 314 reference standard tests subsequently avoided. The AADD cut off had comparable sensitivity to the current cut off despite this increase in specificity; sensitivities for the diagnosis of DVT were 99.28% (95% CI 96.06–99.98%) and 97.72% for PE (95% CI 91.94% to 97.72). There were 3 false negative results using the AADD strategy.

**Conclusions:**

In patients with suspected VTE with a low or moderate pre-test probability, the application of AADD appears to increase the proportion of patients in which VTE can be excluded without the need for reference standard imaging. This management strategy is likely to be associated with substantial reduction in anticoagulation treatment, investigations and cost/time savings.

## Background

Venous thromboembolism (VTE) is a composite term to include deep vein thrombosis (DVT) and pulmonary embolism (PE) and remains a major global disease burden and a leading cause of death and disability [1]. In the UK, the cost of incident events of DVT and PE are estimated at over £640 million [2].

The annual incidence of VTE is approximately 1/1000, increasing with advancing age [3]. Whilst DVT is often well tolerated with early intervention and management, PE is a life-threatening complication of DVT and it is estimated that 10% of patients will die within three months following diagnosis [4, 5].

Definitive diagnosis of VTE is achieved using reference standard diagnostic testing: for DVT this is compression ultrasonography (CUS) of the leg, and for PE this is computed tomographic pulmonary angiography (CTPA) [3, 4]. The decision as to which patients carry sufficient risk to justify these tests involves clinical judgment and validated pre-test probability (PTP) tools such as the two- level dichotomised Wells score [6].

Reliance on reference standing testing can lead to over investigation in patients with suspected VTE and attributable harm. These harms include opportunity costs, wasted patient/clinician time, exposure to radiation and bleeding risk following anticoagulation treatment. The balance of risk and benefit is challenging [7].

If the clinical risk of VTE is low, a simple blood test (D-dimer) is the first line test recommended by multiple international guidelines; a negative result below the threshold is used to exclude VTE [6–12]. When used in this manner, the D-dimer test has high sensitivity but relatively poor specificity, leading to many false positive and increased numbers of patients undergoing unnecessary investigations. This is a particular issue with advanced age. Over the last decade several studies have suggested a potential improvement in specificity using novel higher cut points in the older age group to mitigate these issues [13].

Despite these studies, practice in the UK remains variable. Many emergency departments have not adopted novel strategies and continue to pursue high rates of imaging.

We sought to evaluate the pragmatic performance and implications of using an age adjusted strategy to rule out VTE in an emergency department setting, within a large cohort of patients with suspected disease.

## Methods

### Aims

The aim of this study was to determine the potential effects of applying an age-adjusted D-dimer (AADD) cut point rather than a conventional threshold and evaluate whether adoption of AADD would be a safe and effective alternative to current practice.

The primary outcome was to determine the diagnostic test characteristics of an AADD strategy in all patients with suspected VTE, presenting through the ED. Secondary outcomes included proportional imaging reduction and the potential cost and time savings associated with this strategy.

### Methods

This was single centre retrospective diagnostic cohort study of all patients evaluated for possible VTE who presented to an Emergency Department (ED) in the Northwest of England over a consecutive 12- month period between January and December in 2016. The base sample included patients who had a positive D-Dimer which exceeded the trust cut off point. The department uses a two level modified Wells score as recommended by the National Institute for Health and Care Excellence (NICE) 2020 guidelines [14]. The two level Wells score determines whether VTE is likely or unlikely; in likely cases, clinicians should proceed to imaging, whereas in unlikely cases, it is appropriate to send a D-dimer and await the result. If the D-dimer is below the threshold in these patients then VTE can be excluded.

In the department, we use a local assay reported in ng/ml D-dimer Units (DDU) with a locally suggested threshold of 230 ng/ml. However, in order to assess the test characteristics for AADD, we applied a conversion formula by multiplying the local result in DDU by two to convert into Fibrinogen Equivalent Units (FEU). We then applied a conventional FEU equivalent of cut-off point of 500 ng/ml and AADD cut off point of (age × 10) in FEU. We selected the cut off of 500 ng/ml in the literature rather than using the local cut offs to account for the conversions of units and the literature recommended cut offs [15].

The immunoassay used was Haemosil DDimer HS Kit. 

Patients were identified through pathology D-dimer requests originating from the ED. Clinical assessment records and reference standard test results were reviewed by trained research assistants using an electronic patient record system (EPR). A definitive reference standard imaging result was compiled and coded as VTE positive or negative. Investigations reviewed included compression ultrasonography (CUS) of the leg, CTPA and V/Q imaging modalities.

All cases of disagreement were adjudicated by the senior supervising ED consultant. Clinical records of patients with false negative outcomes were investigated further by an independent panel. Patients with clear prospective documentation recording a high pre-test probability, VTE as the most likely diagnosis, or with eventual diagnosis of chronic thrombus left untreated or superficial venous thrombosis were reported individually but excluded from the final analysis.

#### Inclusion criteria

All patients presenting to the ED over 50 years of age who had a positive D-dimer result exceeding the local trust assay.

#### Exclusion criteria

Patients who did not undergo a reference standard result (D-dimer test result not acted upon). Patients with a D-dimer result below the standard cut point (negative). Patients diagnosed with VTE who had no D-dimer test ordered at any stage.

All statistical analyses were performed using MD Calc. 2016 Statistical Software Version 16.4.3.

All methods were carried out in accordance with relevant guidelines and regulations.

#### Approvals

This project underwent local review and was approved through Research and Innovation as a service evaluation (ref: S20HRANA07). Patient consent was not required.

## Results

**Fig. 1:**
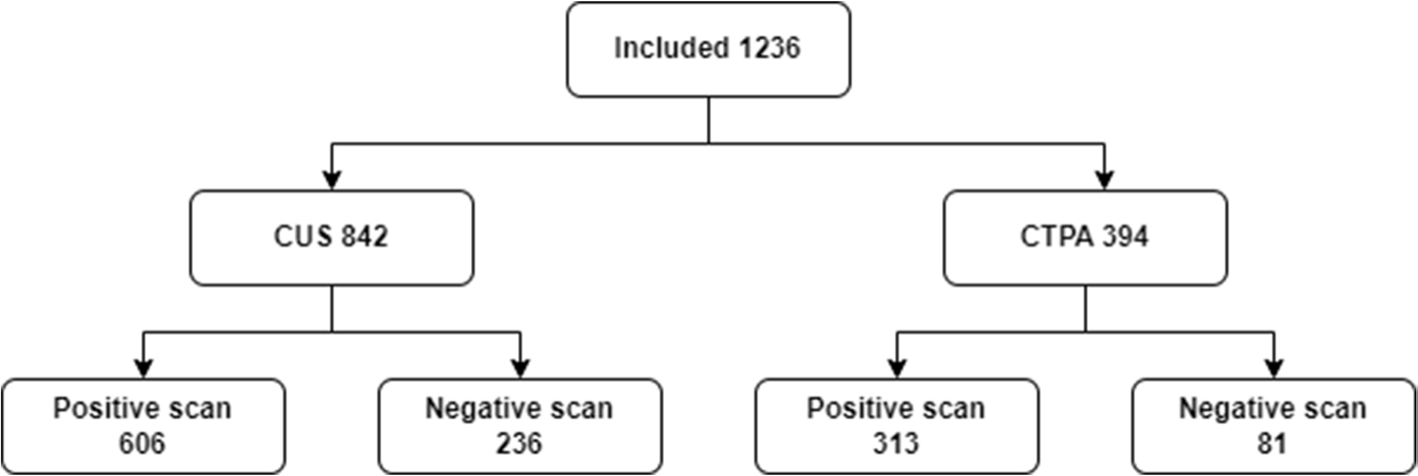
Patient flow through the study

During the study period, 4913 D-dimer tests were requested, 2132 of which yielded positive results above the conventional threshold (DDU of 230 ng/ml was converted to FEU by multiplying by two and a 500 ng/ml used as the cut-off point). One thousand two hundred thirty-six patients received reference standard investigations and were therefore suitable for inclusion in the study. The patient flow through the study is illustrated in Fig. 1.

Twenty potential false negatives were identified (patients with a negative AADD but a positive reference standard test). These are outlined in Table 1. Following an independent panel review, 17 of these were identified as having limited clinical relevance and were excluded as per previous criteria, the common reasons for exclusion included high pre-test probability, non- occlusive thrombosis and chronic thrombus. Three out of the 20 cases were identified as false negatives and are outlined in Table 2. These cases would potentially have been missed in clinical practice, if an AADD strategy had been applied. Each of these patients went on to receive treatment with therapeutic dose anticoagulation, except one.

**Table 1:** Screening of potential false negatives

Patient Age	D-dimer result (FEU)	Age Adjusted Cut off (age × 10) (FEU)	Well’s Score	Reference Standing Imaging	Decision
91	572	910	Not calculated (however DVT was listed as number listed differential)	CUS- partial non occlusive common femoral vein DVT	Exclude- high clinical suspicion and non-occlusive thrombus
89	496	890	6	CTPA- VTE positive	Exclude- high pre-test probability
85	622	850	Not calculated. However high risk as previous DVT	CUS- VTE positive	Exclude- non occlusive thrombus
85	654	850	2	CUS- small non occlusive thrombus suggested	Exclude- non occlusive thrombus
81	592	810	Not calculated. Immediately post operatively	CUS– non occlusive chronic thrombus	Exclude – chronic thrombus
76	642	760	Not calculated. 3 week history of thrombophlebitis	CUS- positive for thrombophlebitis	Exclude- thrombophlebitis
75	300	750	Not calculated	CUS- non occlusive chronic thrombus	Exclude- chronic thrombus
75	544	750	5	CUS- VTE positive	Exclude- high pre-test probability
77	704	770	Not calculated. 4 from data available	CUS- VTE positive	Exclude- high pre-test probability
53	504	530	Not calculated	CTPA – VTE positive	Include as false negative
46	462	460	Not calculated	CUS- VTE positive, however repeat scan negative	Exclude patient < 50 so normal cut off would be used
73	670	730	Not calculated. 2–3 day history of swollen ankle. Received subcutaneous thromboplastin	CUS- VTE positive	Exclude – high pre-test probability
84	634	840	Not calculated. No data available	CTPA- VTE positive	Include as false negative
84	628	840	Not calculated No data available	CTPA- VTE positive	Exclude- repeat CTPA in the above patient during same admission
60	600	600	-1	CUS- VTE positive	Include as a false negative
88	480	880	2, high clinical suspicion	CUS- VTE positive	Exclude- high pre-test probability
84	764	840	3	CUS- VTE positive	Exclude- high pre-test probability
83	692	830	3	CUS- VTE positive	Exclude- high pre-test probability
76	656	760	Not calculated	CUS- partial non occlusive thrombus in the superficial femoral vein – not treated	Exclude- non occlusive thrombus. Not treated
93	546	930	3	CUS- VTE positive	Exclude-high pre-test probability

^*^FEU refers to fibrinogen equivalent units

^*^Age adjusted D-dimer threshold calculation (age in years × 10) in FEU

**Table 2:** Included false negatives

Patient Age	D-Dimer Result (FEU)	Age adjusted cut off (age × 10) (FEU)	Well’s Score	Reference standard imaging
53	504	530	Not calculated	CTPA- VTE positive
84	634	840	Not calculated No data available	CTPA- VTE positive
60	600	600	-1	CUS- VTE positive

A total of 314/1236 (25.1%) results would have been coded as true negative as opposed to false positives if the age adjusted cut off point has been applied. This suggests 314 reference standard tests would have been avoided in 2016 had this strategy been used.

The test characteristics for AADD testing after exclusion of the 17 cases with high pre-test probability or chronic/superficial disease are shown in Table 3.

**Table 3:** Diagnostic test characteristics for applying Age Adjusted Threshold after exclusions

Statistic	95% Confidence Interval (all VTE)	95% Confidence Interval (DVT only)	95% Confidence Interval (PE only)
Sensitivity	98.67% (96.17–99.73)	99.28% (96.06–99.98)	97.70% (91.94–99.72)
Specificity	31.09% (28.24- 34.05)	33.43 (29.95–37.05)	25.73% (20.94–31.01)
Positive likelihood ratio	1.43 (1.37–1.50)	1.49 (1.41–1.57)	1.32 (1.22–1.42)
Negative likelihood ratio	0.04 (0.01–0.13)	0.02 (0.00–0.15)	0.09 (0.02–0.36)
Positive predictive value	24.4% (23.46- 25.09)	22.77% (21.83–23.74)	27.16% (25.73–28.63)
Negative predictive value	99.00% (97.13–99.69)	99.58% (21.83–23.74)	97.53% (90.83–99.37)
Disease prevalence	18.28 16.17–20.55	16.51 (14.06–19.19)	22.08 (18.08–26.51)

More patients with positive D-dimer results were investigated for DVT than for PE, with 842 compression ultrasonography (CUS) of the leg, compared to 394 CTPA scans. The use of age adjusted cut point was more sensitive in patients being investigated for DVT, with a sensitivity of 99.28% (95% CI 96.06%-99.98%) compared to 97.7% (95% CI 91.94% to 99.72%) for those investigated for PE. Specific reference ranges for the DVT and PE cohorts are shown in Table 3.

## Discussion

### Principal findings

Our results suggest that the use of an AADD cut point for patients > 50 has comparable sensitivity to that of the current cut off for suspected VTE. In addition, application of this cut point would have led to an annual saving of 235 compression ultrasonography (CUS) of the leg and 79 CTPAs in a calendar year. In the climate of reduced bed capacity, increased demand and stress on a resource limited system, our work has the potential to coincide with the move towards enhanced ambulatory care [4]. In our study, the estimated cost saving based on a compression ultrasonography (CUS) of the leg costing £77.19 and a CTPA £106.2 would be (£18,139.65 and £8389.8) respectively, leading to a potential total cost saving of £26,529.45, if the AADD strategy had been applied [16, 17].

### Strengths and limitations

The study benefits from a large sample size over a 12-month period and was a pragmatic evaluation of a busy tertiary centre NHS service. Current evidence has previously focused on PE, our study additionally looked at the effects on DVT. The retrospective nature of this study was the main limitation. Authors relied upon real time documentation and variations in accuracy of documentation may have led to inconsistency. In contentious cases, the Wells score was calculated retrospectively which may not have been accurate. The data sets were studied by different investigators, without duplication or cross check. Whilst every effort was made to ensure a standardised data collection, variations and human error cannot be ruled out.

Of the 2132 positive D-dimer results, 1236 patients received reference standard testing. Hence, a large proportion of the positive D-dimer result were not acted upon by clinicians. Whilst probably an accurate reflection of clinical practice and discretion, it is impossible to say whether these patients would have been VTE positive. No negative D-dimers were studied as the focus was on AADD. In turn, we were unable to make direct comparison to the data characteristics for the current cut off.

Small reductions in negative predictive value could result in clinical cases with significant morbidity, and this must be balanced against the iatrogenic harms of a lower threshold/more aggressive investigation policy, such as incidental findings, radiation exposure, unnecessary anticoagulation and contributions to hospital crowding.

Multiple quantitative assays are in use for D-dimer measurement, with little harmonisation or standardisation. This may affect the validity of the study.

### Comparison with other literature

Our results are in line with previous research. Schouten et al. conducted a systematic review of 12, 497 patients with a low PTP for VTE and found use of AADD increased specificity by 20.5% without any significant reduction in sensitivity [18]. Jaconelli et al. retrospectively reviewed 1649 patients and concluded there would be a 17% increase in negative D-dimer’s if AADD was applied to those > 50, in comparison to the 25.1% in our study [19]. Our study also reported a higher sensitivity of 98.71% compared to 95%. Implementation studies using an AADD strategy have also produced convincing results. The multicentre prospective ADJUST-PE study by Righini et al. included 3346 patients across 19 EDs with suspected PE. Their use of an AADD in those > 50 resulted in PE being safely excluded without a single false negative result in an additional 23.3% of patients [13].

In addition to the concept of age adjusted D-Dimers, the PEGeD study in 2019, prospectively investigated adjusting the cut off to 1000 ng/ml in patients with low clinical PTP. In the low C-PTP group, authors report a relative reduction in the requirements for reference standard imaging of a third (from 51.9% for the conventional strategy, down to the 34.3% with the PEGeD approach) [20]. These findings support the concept of a raised D -Dimer threshold for patients with low pretest probability.

Our results compare to Wells et al. study which assessed the test characteristics of the D-dimer alone in patients deemed to low risk (*n*= 315) based on clinical decision tool- Well’s score. Our negative predictive value 99.00% (97.13–99.69) based on AADD strategy is comparable to the D-dimer alone strategy 99.1% (96.7–99.9) however the AADD strategy has a higher positive predictive value 24.4% (23.46- 25.09) than D-dimer alone 14.1% (7.95–22.6), suggesting that the AADD may be advantageous in diagnosing VTE in patients with a D-dimer above the AADD threshold without reductions in negative predictive value. There are numerous modifications of the diagnostic assessment of patients with suspected VTE including clinical probability, D-dimers, imaging alone and a combination of all these contributing factors [20].

The use of D-Dimer assays to exclude VTE in patients with concurrent COVID-19 remains relatively under investigated and use of age adjusted D-Dimer has not been validated in this setting. Some retrospective analysis suggests the same validated threshold for non-COVID-19 patient should be used to exclude PE in patients with COVID-19 [21], and other suggests that age adjusted D-Dimers may improve diagnostic assessment for patients with PE [22]. Further investigation of the utility of the AADD in the COVID-19 patient population and methods to improve the performance of the tests is required.

NICE 2020 and European Society of Cardiology (ESC) guidance now encourages use of age adjusted D-dimer testing in the diagnosis and management of PE [6].

## Conclusion

In patients with suspected VTE with a low pre-test probability, the application of an AADD appears to safely increase the proportion of patients in which VTE can be excluded without the need for reference standard imaging.

## Data Availability

The datasets generated and/or analysed during the current study are not publicly available due to the internal nature of the service evaluation, but are available from the corresponding author on reasonable request.
